# Medicinal Uses of Coffee

**Published:** 1890-05

**Authors:** 


### MEDICINAL USES OF COFFEE.

It is said that the first use of coffee by man was made by the prior of a convent. He was told by a goatherd of the exciting effect of the berries when eaten by his goats, so he thought he would try them, and see if he could not keep his monks awake during their devotions. He succeeded admirably, and brought coffee into the way of earning its world wide reputation. The most active principle of coffee is caffein; it contains also certain oils which no doubt have a share in its action. Nearly two score years ago a claim was made that green or unroasted berries had a great value in liver and kidney troubles. By one physician they have been used very extensively, and he is quite enthusiastic over them in that class of diseases. He prefers a mixture of two parts Mocha with one part Martinique and Isle de Bourbon coffee. He puts about three drachms of this in a tumbler of cold water, and lets them stand and infuse over night. The next morning, after straining, the infusion is taken on an empty stomach the first thing after getting up. This physician cites many cases of kidney and liver colics, diabetes, nervous headache, etc., which, although rebellious to all other treatment for years, soon yielded to the green coffee infusion. The remedy is a very simple one and certainly worth a trial. Another use of coffee medicinally is in nausea and retching. For that purpose a strong infusion is made of the berries which have been ground and roasted ; and it is sipped while very hot. This oftentimes acts exceedingly well ; and rather better if a strong mustard plaster is applied to the pit of the stomach at the same time.

